# Effectiveness of Calcium Sulfate and Hydroxyapatite Composite in Collapse Prevention in Osteonecrosis of Femoral Head

**DOI:** 10.7759/cureus.24408

**Published:** 2022-04-23

**Authors:** Javed Jameel, Siddhartha Sinha, Arvind Kumar, Owais A Qureshi, Sandeep Kumar, Neel Aggarwal, Anmol Dua, Mohd Junaid Nagori, Rizwan Khan

**Affiliations:** 1 Orthopaedics, Hamdard Institute of Medical Sciences and Research, New Delhi, IND

**Keywords:** osteonecrosis, hydroxyapatite, hip joint, femoral head, core decompression, calcium sulfate, bone-graft, bioceramic, avascular necrosis

## Abstract

Introduction

Calcium-sulfate-hydroxyapatite bioceramics have been widely used as void fillers in bone. However, their effectiveness as void fillers in core decompression for osteonecrosis of the femoral head (ONFH) in preventing femoral head collapse prevention has limited evidence. The current study investigates the effectiveness of calcium-sulfate-hydroxyapatite bioceramics as a void filler in the core decompression procedure for ONFH.

Methods

We retrospectively reviewed the clinical and radiological records of ONFH patients that underwent core-decompression using either autologous iliac crest cancellous bone graft or calcium-sulfate-hydroxyapatite bioceramic paste as void fillers with at least one-year follow-up. The primary outcome of this study was the radiological progression of collapse in the last available standard anteroposterior (AP) radiographs of the hip. The collapse progression was compared between the two groups based on void fillers.

Results

This study included patient records with 44 hip joints that underwent core decompression. There were five female and 33 male patients. The mean age was 29.1±6.3 years. The mean follow-up duration was 21.4±3.4 months. No significant differences in collapse progression were observed between the two groups based on void fillers.

Conclusion

The use of calcium-sulfate-hydroxyapatite as a void filler in core decompress for ONFH is not superior to the autologous cancellous bone in terms of collapse prevention and mechanical support. Further modifications in the core decompression techniques and well-planned prospective studies would help establish sound recommendations.

## Introduction

Prevention of collapse has a prognostic role in managing osteonecrosis of the femoral head (ONFH) [1]. Classically, cancellous bone grafts have been used to fill the voids resulting from core decompression in ONFH [2]. The major concern with these grafts is the lack of structural support, their shrinkage with time, and donor site morbidity. The former two factors can contribute to the collapse progression of the osteonecrosis zone. Calcium-sulfate-hydroxyapatite bioceramics have been widely used as void fillers in bone [3]. They also form the basis of advanced core decompression in which the void is filled with a bioceramic paste [4]. It has been suggested that calcium sulfate acts as a resorbable carrier for hydroxyapatite, and hydroxyapatite is highly osteoconductive, promoting bone ingrowth [5]. Previously, calcium sulfate alone and its composite mixture with calcium phosphate have been used to address voids in core decompression [6-8]. While functional outcomes have been shown to improve with the use of bone graft substitutes, the evidence of collapse progression has not been adequately studied. Few studies have analyzed the effect of calcium sulfate and calcium phosphate in collapse prevention in ONFH, but the results have not been satisfactory [6,8]. Limited evidence suggests the better performance of calcium hydroxyapatite alone without calcium sulfate in collapse prevention [9]. However, this has not been much investigated.

Similarly, the effectiveness of advanced core decompression using calcium-sulfate-hydroxyapatite bioceramics to prevent collapse prevention has not been adequately studied. Therefore, the current study investigates the effectiveness of calcium-sulfate-hydroxyapatite bioceramics as a void-filler in core decompression procedures for ONFH.

## Materials and methods

After approval from the institutional review board, we retrospectively reviewed the clinical and radiological records of all ONFH patients that underwent core-decompression using either autologous iliac crest cancellous bone graft or calcium-sulfate-hydroxyapatite bioceramic paste, from January 2016 onwards with at least one-year follow-up. The study included cases operated between Jan 2016 to Dec 2019. Based on the available information, the inclusion criteria were idiopathic types of ONFH in pre-collapse stages (Ficat Arlet I and IIB) [10], considering that secondary types could have variable collapse progression. Exclusion criteria were the post-collapse stages (Ficat Arlet IIB and higher) and non-idiopathic types of ONFH with risk factors like smoking, alcoholism, coagulopathies, trauma, infection, post-surgical cases, etc. The primary outcome of this study was the radiological progression of collapse in the last available standard anteroposterior (AP) radiographs of the hip. We used the concentric circle technique described by Kumar et al. [11] to measure the proportionate width of the deficient zone of the femoral head in preoperative and last available follow-up radiographs after one year of core decompression. The technique measured the deviation of the femoral head from a circular contour in AP radiographs of the hip joint. The measurements were made by two orthopedic residents who were trained in the software-based measurements of radiographic parameters. An increase in the sequential width of the deficient zone between the preoperative and follow-up radiographs was marked as the collapse progression.

We measured the baseline demographic characteristics, such as gender distribution, age, stage of ONFH based on Ficat Arlet classification [10], and follow-up duration. We compared these parameters between the two groups based on the type of void-filler during core decompression. The categorical variables were compared between the two groups using the chi-square test, and the continuous variables were compared using an unpaired t-test. The continuous variables measurements were expressed as mean±standard deviation, and the categorical variables were expressed as proportions. The femoral head collapse in preoperative and follow-up radiographs within each group was compared using a paired sample t-test. The collapse progression was compared between the two groups using an unpaired sample t-test. A p-value of less than 0.05 was considered statistically significant.

## Results

A total of 38 patient records with 44 hip joints that underwent core decompression were reviewed. There were five female and 33 male patients; 32 hip joints belonged to Ficat Arlet Stage I ONFH, and the remaining were stage II cases. The mean age was 29.1±6.3 years. The mean follow-up duration was 21.4±3.4 months. No significant differences in demographic characteristics were observed between the two groups based on the type of void-filler material. The preoperative values of femoral head deficiency were comparable among the two groups. While the last follow-up values of femoral head deficiency were higher in the cancellous bone-graft group, the change in the femoral head deficiency compared to the preoperative values was statistically insignificant between the two groups. The detailed results are presented in Table 1.

**Table 1:** Comparison of baseline demographic and radiological characteristics among the two void-filler-based groups.

Characteristics	Cancellous bone-graft group	Calcium-sulfate-hydroxyapatite paste group	Remarks
Male-female distribution (number of patients)	16 males, 2 females	17 males, 3 females	No significant difference (p = 0.72)
Mean age (in years)	29	29.2	No significant difference (p = 0.46)
Ficat Arlet stages distribution (number of hip joints)	Stage I: 14, Stage II: 7	Stage I: 18, Stage II: 5	No significant difference (p = 0.38)
Preoperative femoral head deficiency (%)	0.7±0.9	0.5±0.9	No significant difference (p = 0.13)
Follow-up duration (months)	21.2±3.2	21.6±3.5	No significant difference (p = 0.36)
Last follow-up femoral head deficiency (%)	4.8±1.9	3.9±1.4	Significant difference (p = 0.03)
Collapse progression (%)	3.95±1.2	3.41±1.4	No significant difference (p = 0.07)

## Discussion

The findings of the current series suggest that calcium-sulfate-hydroxyapatite void fillers do not offer any major advantage over autologous cancellous bone grafts in terms of collapse progression. While they have the advantage of nil graft donor-site morbidity, the high costs can limit its overall effectiveness for use among all patients. At our institute, we offered both options to the patients since the calcium-sulfate-hydroxyapatite-based bone-graft substitutes were introduced as void fillers. The patient chose the void filler option according to financial status, willingness for iliac crest bone graft, previous history of bone graft substitute allergy, and are often based on their personal choices. The groups in our study were comparable in terms of baseline demographic characteristics and preoperative femoral head deficiencies. Therefore, the measured outcomes are potentially reliable. The outcomes suggest that the theoretical advantage of the structural support provided by the bioceramic void filler may not correlate with the equivalent collapse prevention of the ONFH lesion.

We used the modified core decompression technique for ONFH described by Landgraeber and colleagues [4]. The technique involves debridement of the necrotic cavity using an expandable reamer, and the cavity is either filled with cancellous graft or injectable calcium-sulfate-hydroxyapatite composite. An empty cavity remains at a mechanical disadvantage, and a void-filler helps support it [4]. The literature concerning the effectiveness of calcium-sulfate-hydroxyapatite composite in core decompression is limited and heterogeneous. While early reports suggested satisfactory radiological outcomes, later reports suggested significant progression of femoral head collapse despite filling the void with the bioceramic [4,8,12,13]. In addition, some patients have local soft-tissue tissue reactions due to exposure to calcium sulfate [14]. Hydroxyapatite is a highly osteoconductive material but with no osteogenic properties. There have been some modifications to the advanced core decompression [14]. Landgraeber et al. [8] filled the necrotic cavity of ONFH with core-derived cancellous bone and impacted that with calcium-sulfate-hydroxyapatite composite. Lin et al. [15] used autologous cancellous bone mixed with allogeneic fibular graft for filling the necrotic cavity and the core-derived cancellous bone to impact it. Recently, Classen et al. [13] suggested no major improvement by using a resorbable and osteoinductive bone-graft substitute for advanced core decompression.

While modifications have been happening, whether these techniques add any advantage to the advanced core decompression procedure has not been established. In our study, the mean follow-up is approximately 21 months, sufficient for the resumption of day-to-day activities. The impending collapse can be evident after such duration and can suggest the effectiveness of void filler material. Our findings support the overall evidence suggesting no major advantage of the calcium-sulfate-hydroxyapatite mixture over the conventional cancellous bone grafts, except for the graft site morbidity.

There are some major limitations of the current study. First, the study focuses on the mechanical aspect of void fillers in ONFH and not on the functional outcomes. However, it is difficult to correlate symptoms with the femoral head shape as many osteoarthritic patients are asymptomatic or only mildly symptomatic. Second, several other factors related to patient profile, surgical technique, and compliance to postoperative rehabilitation can affect the quality of radiological outcomes. While we considered only the idiopathic cases as per records, additional factors can better be predicted through a prospective study. Third, the series presents a mean follow-up of approximately 21 months. The long-term outcomes and the need for total hip replacement can't be predicted through this study. Fourth, the study has a small sample size of 38 patients. The sample size was small considering that we excluded all cases of post-collapse stages. Thus, a large sample-based study would probably contribute to more reliable conclusions. The evidence presented current study could trigger further such studies. Lastly, the radiographic progression of collapse does not predict the contribution of void filler to the healing of osteonecrosis. A magnetic resonance imaging (MRI) study would better investigate such correlation. Nevertheless, the study contributes to the literature that is yet to achieve consensus regarding using bio-substitutes as void filler in core decompression for ONFH.

## Conclusions

Calcium sulfate hydroxyapatite’s use as a void filler in core decompression for ONFH is not superior to the autologous cancellous bone in terms of collapse prevention and mechanical support. It may be offered as an option to prevent bone-graft donor site morbidity and as per patient demand. Further modifications in the core decompression technique and well-planned prospective studies with a larger sample size would help establish sound recommendations.
